# Stringent Emission Control Policies Can Provide Large Improvements in Air Quality and Public Health in India

**DOI:** 10.1029/2018GH000139

**Published:** 2018-07-03

**Authors:** Luke Conibear, Edward W. Butt, Christoph Knote, Stephen R. Arnold, Dominick V. Spracklen

**Affiliations:** ^1^ Engineering and Physical Sciences Research Council Centre for Doctoral Training in Bioenergy University of Leeds Leeds UK; ^2^ Institute for Climate and Atmospheric Science, School of Earth and Environment University of Leeds Leeds UK; ^3^ Meteorological Institute LMU Munich Munich Germany

**Keywords:** ambient air pollution, health risk, India, disease burden, air pollution control pathways, emission scenarios

## Abstract

Exposure to high concentrations of ambient fine particulate matter (PM_2.5_) is a leading risk factor for public health in India causing a large burden of disease. Business‐as‐usual economic and industrial growth in India is predicted to increase emissions, worsen air quality, and increase the associated disease burden in future decades. Here we use a high‐resolution online‐coupled model to estimate the impacts of different air pollution control pathways on ambient PM_2.5_ concentrations and human health in India. We find that with no change in emissions, the disease burden from exposure to ambient PM_2.5_ in 2050 will increase by 75% relative to 2015, due to population aging and growth increasing the number of people susceptible to air pollution. We estimate that the International Energy Agencies New Policy Scenario (NPS) and Clean Air Scenario (CAS) in 2050 can reduce ambient PM_2.5_ concentrations below 2015 levels by 9% and 68%, respectively, offsetting 61,000 and 610,000 premature mortalities a year, which is 9% and 91% of the projected increase in premature mortalities due to population growth and aging. Throughout India, the CAS stands out as the most effective scenario to reduce ambient PM_2.5_ concentrations and the associated disease burden, reducing the 2050 mortality rate per 100,000 below 2015 control levels by 15%. However, even under such stringent emission control policies, population growth and aging results in premature mortality estimates from exposure to particulate air pollution to increase by 7% compared to 2015, highlighting the challenge facing efforts to improve public health in India.

## Key Points

- Air pollution is a major risk factor for human health in India
- Population aging and growth will increase the disease burden due to exposure to particulate air pollution even under no emission change
- Stringent emission control reduces mortality rate in 2050 below 2015 levels although total premature mortality increases

## 1 Introduction

A recent India‐specific Global Burden of Diseases, Injuries, and Risk Factors Study (GBD) identified air pollution as a leading risk factor for public health (India State‐Level Disease Burden Initiative Collaborators, 2017; Indian Council of Medical Research et al., 2017). Exposure to ambient particle mass with aerodynamic diameter less than 2.5 μm (particulate matter 2.5, PM_2.5_) causes 1 million premature mortalities per year in India, where it is currently the second leading risk factor contributing to mortality (Cohen et al., 2017; Conibear et al., 2018; GBD 2016 Risk Factors Collaborators, 2017). The Indian population is currently exposed to very high ambient PM_2.5_ concentrations (Conibear et al., 2018; Ministry of Environment and Forests, 2018), with annual mean concentrations of up to 150 μg/m^3^ in the Indo‐Gangetic Plain (IGP) and episodic concentrations regularly reaching 800 μg/m^3^. These concentrations are 15 and 32 times larger than the World Health Organization (WHO) Air Quality Guidelines (AQG) respectively (World Health Organization, 2006).

Future large growth in the Indian economy and energy consumption is projected to increase emissions substantially under a business‐as‐usual scenario relative to the present day (GBD MAPS Working Group, 2018), with PM_2.5_, sulfur dioxide (SO_2_), and nitrogen oxides (NO_x_) emissions approximately doubling by 2050 relative to 2015 (International Energy Agency, 2016b; Sharma & Kumar, 2016), increasing PM_2.5_ concentrations by 67% (Pommier et al., 2018). Climate change is also predicted to alter ambient PM_2.5_ concentrations in India; however, these changes are smaller relative to emission changes (Fang et al., 2013; Jacobson, 2008; Kumar et al., 2018; Pommier et al., 2018; Silva et al., 2017). In addition to changing concentrations, the disease burden from air pollution exposure is affected by population growth, population aging, and changes in baseline mortality rates (Hughes et al., 2011; Lelieveld et al., 2015). These other drivers vary the number of people susceptible to air pollution, and their effects can outweigh the impact from emission changes (Chowdhury et al., 2018; GBD MAPS Working Group, 2016, 2018). The business‐as‐usual scenario in India is predicted to increase estimates of premature mortality from ambient PM_2.5_ exposure (Anenberg et al., 2012; GBD MAPS Working Group, 2018; International Energy Agency, 2016a; Lelieveld et al., 2015).

Alternative air pollution control pathways (scenarios) for India have been developed and evaluated in previous studies (GBD MAPS Working Group, 2018; International Energy Agency, 2016a, 2016b; Pommier et al., 2018; Sharma & Kumar, 2016). The International Energy Agency (IEA) developed the New Policy Scenario (NPS), which considers all relevant existing and planned policies as of 2016, and the Clean Air Scenario (CAS), which represents aggressive policy action using proven energy policies and technologies tailored to national circumstances (International Energy Agency, 2016a, 2016b). The NPS in 2040 was found to offset most of the growth in emissions of SO_2_, NO_x_, and PM_2.5_ relative to 2015 bringing the mean total growth per pollutant down to 9%, while under the CAS in 2040, emissions were reduced below 2015 levels by an average of 65% (International Energy Agency, 2016a, 2016b). The associated disease burden due to this change in ambient air pollution exposure varied by +53% and ‐5% for the NPS and the CAS scenarios, respectively, where population growth and aging to 2040 substantially increased the number of people susceptible to air pollution (International Energy Agency, 2016a). A recent study of Indian air quality and associated disease burden by the GBD MAPS Working Group analyzed a business‐as‐usual reference scenario, an ambitious scenario reflecting stringent emission standards, and an aspirational scenario, all through to 2050 (GBD MAPS Working Group, 2018). The GBD MAPS Working Group study estimated population‐weighted ambient PM_2.5_ concentrations across India in 2050 under the reference, ambitious, and aspirational scenarios will change by +43%, +10%, and −35%, respectively, relative to the reference scenario in 2015. The corresponding change in total annual premature mortality from ambient PM_2.5_ exposure in 2050 relative to 2015 will increase under the reference, ambitious, and aspirational scenarios by 234%, 194%, and 125%, respectively, highlighting the strong impact of the demographic transition in India. The ambitious and aspirational scenarios reduced the 2050 reference population‐weighted ambient PM_2.5_ concentrations by 23% and 54% in 2050, respectively, offsetting the increase in annual deaths by 0.34 and 1.2 million, respectively (GBD MAPS Working Group, 2018). Methane (CH_4_) and black carbon mitigation measures have been found to lower future PM_2.5_ concentrations in India, reducing the exposure‐related associated disease burden (Anenberg et al., 2012).

Previous studies that evaluated Indian scenarios (GBD MAPS Working Group, 2018; International Energy Agency, 2016a, 2016b; Pommier et al., 2018) used relatively coarse spatial resolution (0.5° × 0.5° or 0.5° × 0.67°) chemical transport models to estimate the impacts on PM_2.5_ concentrations per scenario, where the GBD MAPS Working Group then applied the fractional impacts on higher resolution ambient PM_2.5_ concentrations to estimate the impacts on health. Model simulations using emissions at these resolutions have been shown to have discrepancies relative to observations (Moorthy et al., 2013; Pan et al., 2015; Pommier et al., 2018), while higher resolution models have been found to produce PM_2.5_ concentrations in closer agreement to observations (Conibear et al., 2018; Kumar et al., 2015; Saikawa et al., 2017). Previous studies analyzing the current contributions of different emission sources in India found residential energy use (RES) emissions to dominate, with substantial contributions from power generation (ENE), industry (IND), and land transport (TRA) Conibear et al., 2018; GBD MAPS Working Group, 2018; Lelieveld et al., 2015; Silva et al., 2016). Ambient PM_2.5_‐related premature mortality in India has been found to be responsive to reductions in SO_2_ emissions, with little sensitivity to NH_3_ emissions (Lee et al., 2015).

In this study, we complement previous work by analyzing multiple air pollution control pathways (scenarios) in India using a higher resolution (30 km, 0.3° horizontal) regional numerical weather prediction model online‐coupled with atmospheric chemistry, with the latest exposure‐response functions (GBD2016) and disease‐specific baseline mortality rates for 2015 and 2050, to make the first high‐resolution analysis of the impacts of scenarios on ambient PM_2.5_ concentrations and resulting disease burden in India. We explore the impact of both the NPS and CAS scenarios from the IEA (International Energy Agency, 2016a). To help interpret the impacts of these scenarios, we conduct idealized simulations where we individually change emissions for the four emission sectors that contribute most to ambient PM_2.5_ concentrations in India (RES, ENE, IND, and TRA). For each sector we conduct simulations with small (−10% and +10%) emission changes as well as a simulation where the emission sector has been completely removed. We assume that both climate and emissions from countries outside India remain unchanged, allowing us to isolate the impacts of changing Indian emissions. We then perform further sensitivity studies to explore the impacts of the Indian demographic and epidemiologic transition through to 2050 on the public health burden associated with air pollution exposure. By conducting a large ensemble of simulations across sectors and scenarios, and estimating resulting ambient PM_2.5_ concentrations and human health impacts, we aim to produce a valuable resource to help inform environmental policy decisions at the state and national levels.

## 2 Methods

### 2.1 Model Description

This study uses the Weather Research and Forecasting model coupled with Chemistry (WRF‐Chem) version 3.7.1 (NCAR et al., 2015) to simulate surface PM_2.5_ concentrations over India for the whole of the year 2014. The model setup, emission inventories, and model evaluation were discussed in detail in previous work (Conibear et al., 2018). WRF‐Chem is a fully online‐coupled, regional, numerical weather prediction model (Grell et al., 2005). Gas phase chemistry is simulated using the Model for Ozone and Related Chemical Tracers, version 4 (MOZART‐4; Emmons et al., 2010) with several updates to aromatic photochemistry, biogenic hydrocarbons, and other species relevant to regional air quality (Hodzic & Jimenez, 2011; Knote et al., 2014). The Model for Simulating Aerosol Interactions and Chemistry (MOSAIC) scheme (Zaveri et al., 2008) with a simplified description of organic aerosols (Hodzic & Jimenez, 2011) is used for aerosol physics and chemistry with four sectional discrete size bins: 0.039–0.156 μm, 0.156–0.625 μm, 0.625–2.5 μm, and 2.5–10 μm (Hodzic & Knote, 2014). Anthropogenic emissions are from the Emission Database for Global Atmospheric Research with Task Force on Hemispheric Transport of Air Pollution (EDGAR‐HTAP) version 2.2 (Janssens‐Maenhout et al., 2015) at 0.1° × 0.1° horizontal resolution. Biomass burning emissions, including agricultural fires, are from the Fire Inventory from National Center for Atmospheric Research (FINN) version 1.5 (Wiedinmyer et al., 2011). The Model of Emissions of Gases and Aerosol from Nature (MEGAN; Guenther et al., 2006) was used to calculate biogenic emissions online. The Goddard Chemistry Aerosol Radiation and Transport (GOCART) dust scheme with Air Force Weather Agency modifications (Chin et al., 2000) was used to calculate online dust emissions. Details of model setup and parameterizations are shown in Table S1.

### 2.2 Air Pollution Control Pathways

We explore the sensitivity of ambient PM_2.5_ concentrations and the related disease burden to different scenarios. All simulations are annual simulations using meteorology and boundary conditions for the year 2014. We perform simulations with NPS and CAS scenarios from the IEA (International Energy Agency, 2016a). The NPS considers all relevant existing and planned policies as of 2016, including India's Intended Nationally Defined Contribution to greenhouse gases under the United Nations Framework Convention on Climate Change. In the NPS SO_2_, NO_x_, and PM_2.5_ emissions increase overall by 10%, 10%, and 7% in 2040 relative to 2015, respectively. SO_2_ emissions from the power sector are largely reduced by air quality policies such as The New Environment Protection Amendment Rules (Ministry of Environment Forests and Climate Change, 2015). Transport NO_x_ emissions decrease due to the Bharat VI standards reducing emissions from buses and trucks (Ministry of Road Transport and Highways, 2016). Residential PM_2.5_ emissions decrease due to the expansion of liquefied petroleum gas (LPG) promotion policies such as the Pradhan Mantri Ujjwala Yojana (PMUY; Ministry of Petroleum and Natural Gas, 2018a) and the direct benefit transfer of LPG (DBTL) scheme Pratyaksh Hanstantrit Labh (PAHAL; Ministry of Petroleum and Natural Gas, 2018b). Substantial industrial growth offsets these reductions, largely due to increases in iron and steel production using coal with low emission standards, despite Indian coal and imported Indonesian coal having low sulfur contents.

The CAS represents aggressive pollution abatement policies using proven energy policies and technologies. In the CAS SO_2_, NO_x_, and PM_2.5_ emissions decrease overall by 69%, 50%, and 76% in 2040 relative to 2015, respectively. SO_2_ and NO_x_ emissions are lowered primarily due to industrial controls on iron, steel, and cement production, and stricter standards for heavy duty vehicles. PM_2.5_ emission reductions benefit from tight standards in iron and steel manufacturing, in addition to universal access to clean cooking facilities such as modern fuels and clean cookstoves. Both the NPS and CAS suggest that the industrial sector will dominate anthropogenic emissions of SO_2_, NO_x_, and PM_2.5_ in the future.

**Figure 1:**
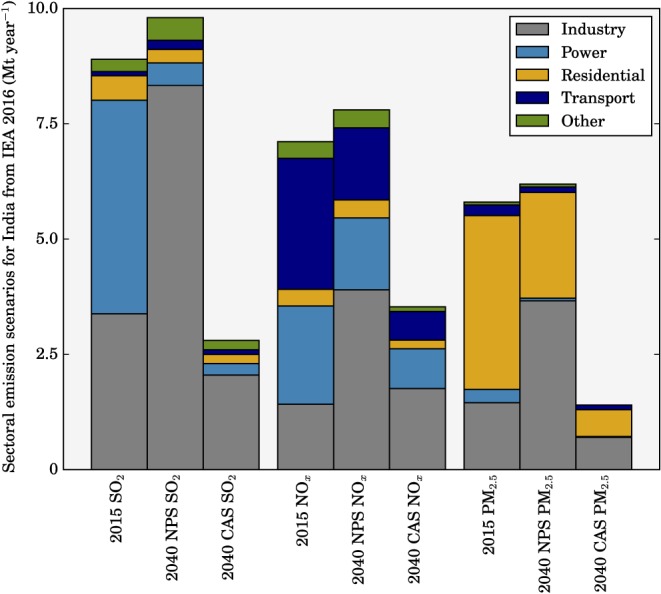
Cumulative emissions per source within India in 2015 and projected emissions in 2040 under the NPS and CAS (International Energy Agency, 2016a).

The NPS and CAS were applied to our anthropogenic emissions by scaling emissions by factors from the IEA (International Energy Agency, 2016a), which are shown in Figure 1 and Table S2. Sector‐specific SO_2_, NO_x_, and PM_2.5_ emissions are scaled by factors from the IEA (International Energy Agency, 2016a). Sector‐specific black carbon and organic carbon emissions are scaled by the same factor as PM_2.5_, while carbon monoxide and all volatile organic compounds are scaled by the mean of total factors across all sectors (1.09 and 0.35 for the NPS and CAS, respectively). We did not scale ammonia (NH_3_) emissions as per previous studies (GBD MAPS Working Group, 2018), due to its low contribution to ambient PM_2.5_ concentrations through the agricultural sector in India (Conibear et al., 2018; Pozzer et al., 2017), the low mortality response from NH_3_ changes in India (Lee et al., 2015; Pozzer et al., 2017), and that the level of NH_3_ emissions are relatively stable with no control measures applied (GBD MAPS Working Group, 2018). To help interpret the results for the CAS and NPS simulations, we perform idealized simulations where each of the leading four emission sources to ambient PM_2.5_ concentrations (RES, ENE, IND, and TRA) previously identified (Conibear et al., 2018) have emissions increased or decreased by 10% in addition to a simulation where emissions from that sector were completely removed.

### 2.3 Model Evaluation

The control simulation was extensively evaluated for air quality and meteorology in previous work (Conibear et al., 2018). Simulations for 2014 were evaluated against surface measurements of hourly PM_2.5_ concentrations for 45 sites across India in 2016 from the Central Pollution Control Board (Ministry of Environment and Forests, 2018) and found the model to be unbiased against the observations and captured the annual mean PM_2.5_ abundances (normalized mean bias = −0.10; Conibear et al., 2018). The model underestimated ambient PM_2.5_ concentrations near the Thar Desert and in the central IGP, which was also found in previous simulations over India (Kumar et al., 2014). Ambient PM_2.5_ concentrations were similar to those from GBD2015 (Shaddick et al., 2018), apart from our model simulating lower estimates in the central and western IGP (Conibear et al., 2018). Further model evaluation for aerosol optical depth against the aerosol robotic network surface measurements showed similar close agreement (normalized mean bias = 0.09; Conibear et al., 2018).

### 2.4 Health Impact Estimation

Long‐term exposure to PM_2.5_ has been found to be a cause of cardiovascular mortality and morbidity, a cause of cancer, and a likely cause of respiratory effects (Brook et al., 2010; Loomis et al., 2013; Newby et al., 2015; U.S. Environmental Protection Agency, 2009). The disease burden associated with long‐term ambient PM_2.5_ exposure was estimated using the nonlinear integrated exposure‐response (IER) functions from the GBD2016 (GBD 2016 Risk Factors Collaborators, 2017) for five causes: ischemic heart disease (IHD), cerebrovascular disease (CEV), chronic obstructive pulmonary disease (COPD), acute lower respiratory infections (ALRI), and lung cancer (LC) (Figure S1). The IER functions from the GBD are used here as epidemiological studies of long‐term exposure to ambient PM_2.5_ concentrations in India are underway but not yet completed (Balakrishnan et al., 2015). The IER functions use age‐specific modifiers for each disease to estimate relative risk (RR) of mortality associated with ambient PM_2.5_ concentrations (equation (1)), where *z* is the ambient PM_2.5_ concentrations and *z*_*cf*_ is the theoretical minimum risk exposure level where no additional risk is assumed for ambient PM_2.5_ concentrations below 2.5 μg/m^3^ (Cohen et al., 2017). The maximum risk is 1 + α, the ratio of the IER at low to high concentrations is β, and the power of the PM_2.5_ concentration is γ (Cohen et al., 2017). Parameter distributions of α, β, and γ from the GBD2016 (GBD 2016 Risk Factors Collaborators, 2017) were sampled for 1,000 simulations to derive the mean IER function with 95% uncertainty intervals (Cohen et al., 2017).

(1) $$\mathrm{RR}\left(z\right)=1+\alpha \times \left(1-\exp\left\{\beta {\left(z-z_{\mathrm{cf}}\right)}^{\gamma }\right\}\right)$$

Premature mortality (*M*) estimates were then calculated as a function of population (*P*), baseline mortality rates (*I*), and the attributable fraction (*AF*) for a specific RR (equation (2)). Population density, population age groupings, and baseline mortality rates for 2015 and 2050 are taken from the International Futures (IFs) integrated modeling system as explained below in section 2.5. Estimates were split into 5‐year age groupings from 25 to 95 years and upward for all diseases, in addition to 0 to 25 years for ALRI.

(2) $$M=P\times I\times \mathrm{AF}=P\times I\times \left(\mathrm{RR}-1\right)/\mathrm{RR}$$

Years of life lost (YLL) for each disease were estimated as a function of premature mortality and age‐specific life expectancy from the standard reference life table from the GBD2016 (Global Burden of Disease Study 2016, 2017b; equation (3)).

(3) YLL=M×LE

Under the control scenario, we estimate total premature mortality due to exposure to ambient PM_2.5_ in India in 2015 as 900,000 (95% uncertainty interval [95UI]: 683,000–1,252,000) per year, the mortality rate as 62 deaths per 100,000 populations and 21,528,000 (95UI: 15,997,000–30,268,000) YLL. This premature mortality estimate total is 9% lower than reported in Conibear et al. (2018) primarily due to the slightly lower risk estimates for cardiovascular diseases from the GBD2016 exposure‐response function relative to the GBD2015. Our mortality estimate is 13% lower than the estimate from the GBD2016 (GBD 2016 Risk Factors Collaborators, 2017), where the difference results from a combination of slightly lower population density from IFs over India, lower baseline mortality rates at higher ages for cardiovascular diseases, and our slightly lower PM_2.5_ concentrations over the central and western IGP (Conibear et al., 2018; Shaddick et al., 2018).

### 2.5 Future Demographics and Baseline Mortality Rates in India

The IFs integrated modeling system (Hughes et al., 2011) baseline scenario (Hughes et al., 2012) was used to derive 2015 and 2050 population density, population age structure, and baseline mortality rates for COPD, IHD, CEV, LC, and ALRI. Figure S2 shows the variation in baseline mortality, population age distribution, and population density for India between 2015 and 2050. Baseline mortality rates for all diseases in India show reductions in 2050 relative to 2015, especially for ALRI, CEV, and IHD where there are substantial decreases. The population age distribution shifts toward older ages, and there is large population growth, particularly across the IGP. Figure S3 shows the variation in baseline mortality, population age distribution, and population density for India in 2015 between IFs (Hughes et al., 2011) and the GBD2016 (GBD 2016 Risk Factors Collaborators, 2017). IF population density for India in 2015 is very similar to the Gridded Population of the World, Version 4 (GPWv4; Center for International Earth Science Information Network and NASA Socioeconomic Data and Applications Center, 2016), and IF population age groupings for India is similar to the population age structure used by the GBD2016 (Global Burden of Disease Study 2016, 2017a). Baseline mortality rates below 65 years of age are similar for all diseases between IFs and GBD2016 (Institute for Health Metrics and Evaluation, 2018), while above 65 years of age IFs has larger values for respiratory diseases (ALRI and COPD) and smaller for cardiovascular diseases (IHD and CEV) and LC relative to the GBD2016. We found in previous work estimating long‐term premature mortality from exposure to ambient PM_2.5_ in India (Conibear et al., 2018) that estimates using state‐specific baseline mortality rates (Chowdhury & Dey, 2016) agreed within 3% of estimates using GBD2015 baseline mortality rates (Institute for Health Metrics and Evaluation, 2018). Shapefiles were used to split disease burden estimates for India at the country and state level (ICF International, 2017).

### 2.6 Uncertainties

Uncertainty intervals at the 95% level (95UI) were estimated through combining fractional errors in quadrature (i.e., square root of the sum of squares) from 2 standard deviations of biweekly mean PM_2.5_ concentrations per grid cell and derived uncertainty intervals for the IER function. Consistent with the GBD project, the toxicity of PM_2.5_ is treated as homogenous regarding source, shape, and chemical composition due to lack of composition‐dependent exposure‐response functions. Recent research studying the health impacts of long‐term ambient PM_2.5_ exposure in China found the IER function to underestimate the RR of premature mortality over the exposure range experienced (Yin et al., 2017), highlighting the need for further research of the exposure‐response function at the high PM_2.5_ concentrations found in developing countries. This study did not consider the Indian disease burden due to exposure to household air pollution from solid fuel use or ambient ozone exposure, which the GBD2016 (GBD 2016 Risk Factors Collaborators, 2017) estimates to be 782,906 (95UI: 652,172–941,484) and 90,253 (95UI: 35,556–145,570) annual premature mortalities, respectively.

The scenario simulations in this study all use the same meteorology inputs and parameterizations, and hence do not include the impacts of climate changes on air quality, although these changes are likely smaller relative to those driven by emission changes (Fang et al., 2013; Jacobson, 2008; Kumar et al., 2018; Pommier et al., 2018; Silva et al., 2017). Consequently, the validity of our results is limited to the impacts from projected emission changes in India and do not include impacts of future climate change or impacts of emission changes outside India. Important areas of future research are to analyze the impacts of climate change and changes to the inflow of emissions from outside India to air quality in India.

Emissions inventories for India have large uncertainties, especially for the IGP (Saikawa et al., 2017). We do not consider emissions from waste burning, which are substantial in India (Kodros et al., 2016); changes in land use; land cover, or biomass burning emissions; or the impact of Indian emissions on the disease burden in other countries (Zhang et al., 2017).

## 3 Results

### 3.1 Impact of Scenarios on Ambient PM_2.5_ Concentrations in India

**Figure 2:**
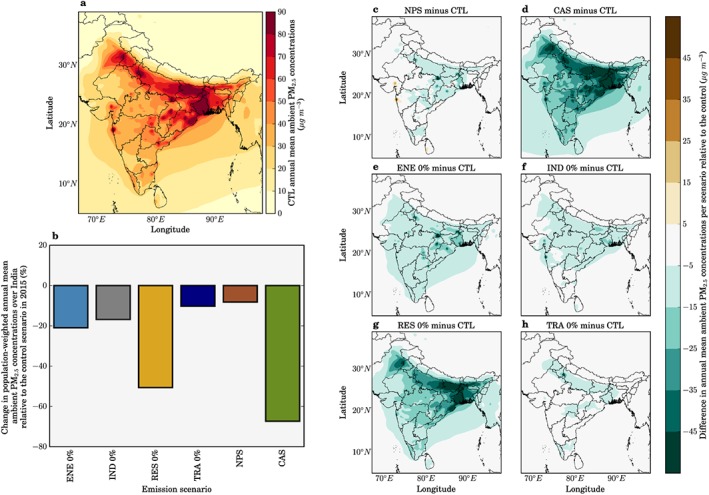
The impact of scenarios on annual mean ambient PM_2.5_ concentrations in India. (a) Annual mean ambient PM_2.5_ concentrations in India in 2015 from the control scenario. (b) National mean changes in 2015 population‐weighted annual mean ambient PM_2.5_ concentrations for each scenario. (c–h) Difference in annual mean ambient PM_2.5_ concentrations for IEA New Policy Scenario (NPS), IEA Clean Air Scenario (CAS), removal of power generation emissions (ENE 0%), removal of industry emissions (IND 0%), removal of residential energy use emissions (RES 0%), and removal of land transport emissions (TRA 0%) scenarios.

Figure 2 shows the impacts of the scenarios on annual mean PM_2.5_ concentrations across India. We find that the NPS and CAS scenarios reduce population‐weighted ambient PM_2.5_ concentrations by 9% and 68%, respectively, relative to the control scenario in 2015. In the CAS scenario, large reductions in ambient PM_2.5_ concentrations are simulated across the IGP, spatially matching the changes simulated by the scenario removing residential energy use emissions (RES 0%). The reduction in ambient PM_2.5_ concentrations achieved by the CAS scenario is greater than the reductions achieved in any of the simulations where emissions from one sector are completely removed.

**Figure 3:**
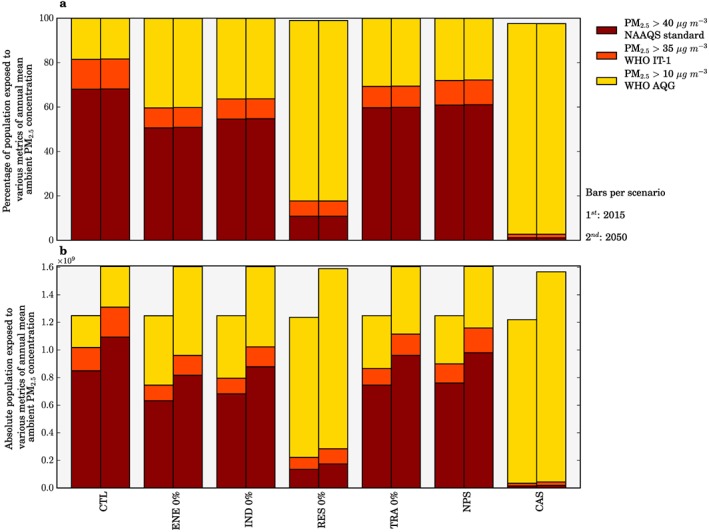
The impact of scenarios on air quality metrics in India. (a) Percentage of population and (b) absolute population in 2015 (first bar) and 2050 (second bar) exposed to annual mean ambient PM_2.5_ concentrations exceeding 10 μg/m^3^ (WHO AQG), 35 μg/m^3^ (WHO IT‐1), and 40 μg/m^3^ (NAAQS) in each scenario.

Figure 3 shows the population exposed to annual mean PM_2.5_ concentrations above the WHO AQG of 10 μg/m^3^, the WHO interim‐target 1 (IT‐1) 35 μg/m^3^, and the Indian National Ambient Air Quality Standards (NAAQS) of 40 μg/m^3^ released by the Government of India (Ministry of Environment and Forests, 2009; World Health Organization, 2006). We find that in all scenarios with both 2015 and 2050 populations, more than 98% of the Indian population remains exposed to annual mean PM_2.5_ concentrations exceeding the WHO AQG. The emission reduction scenarios have a greater impact in bringing population exposure into line with the interim targets, where the percentage of the population in line with the WHO IT‐1 (35 μg/m^3^) is increased from 19% in the control scenario to 97% and 82% for the CAS and RES 0% scenarios, respectively. The increased adherence to these air quality metrics shows that improvements to air quality through emission reductions can provide important public health benefits, which is in agreement with the view from the WHO (World Health Organization, 2006).

### 3.2 Indian Disease Burden Under Air Pollution Control Pathways

**Figure 4:**
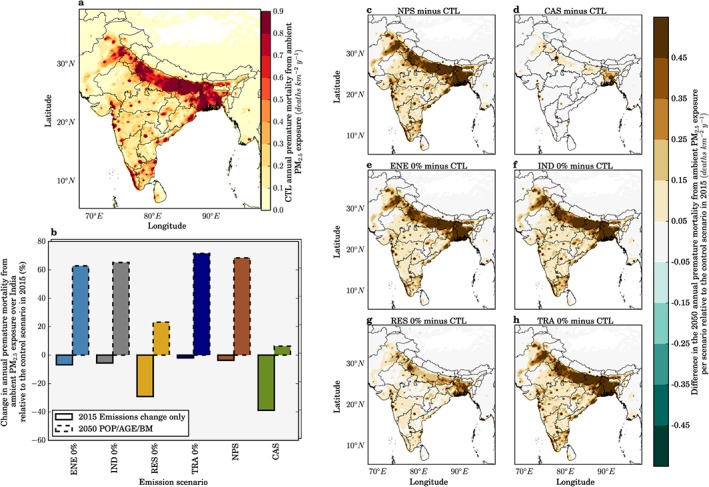
The impact of scenarios on annual premature mortality from exposure to ambient PM_2.5_ in India. (a) Annual premature mortality from exposure to ambient PM_2.5_ in India in 2015 from the control scenario. (b) National mean changes in annual premature mortality estimates from ambient PM_2.5_ exposure per scenario, for both emissions only changes in 2015 and overall changes in 2050. (c–h) Difference in the annual premature mortality from ambient PM_2.5_ exposure in 2050 for IEA New Policy Scenario (NPS), IEA Clean Air Scenario (CAS), removal of power generation emissions (ENE 0%), removal of industry emissions (IND 0%), removal of residential energy use emissions (RES 0%), and removal of land transport emissions (TRA 0%) scenarios relative to the control scenario in 2015.

The impacts of the scenarios on premature mortality estimates from ambient PM_2.5_ exposure across India are shown in Figure 4. Total premature mortality across India is shown assuming population and underlying health data appropriate for both 2015 and 2050 (Figure 4b). Reduced ambient PM_2.5_ concentrations for the NPS and CAS reduce estimates of annual premature mortality for 2015 by 4% and 39%, respectively, relative to the control scenario of 900,000 (95UI: 683,000–1,252,000) annual premature mortalities. Assuming no change in emissions, we estimate total annual premature mortality for the control scenario in 2050 to be 1,577,000 (95UI: 1,210,000–2,209,000). This estimate of premature mortality in 2050 is 75% greater than in 2015 due to population growth and aging, partly offset by reducing baseline mortality rates. Under the NPS and CAS, total premature mortality in 2050 changes by +68% and +7%, respectively, relative to the control scenario estimate for 2015. The NPS and CAS can therefore potentially reduce the annual premature mortality estimate in 2050 by 61,000 and 610,000 deaths relative to the 2015 control, equivalent to offsetting 9% and 91% of the increase in 2050 caused by population aging and growth. Despite the small national increase in premature mortalities (7%), the disease burden is actually reduced in some states (Jammu and Kashmir, Himachal Pradesh, Uttaranchal, and Sikkim; Figure 4d).

**Figure 5:**
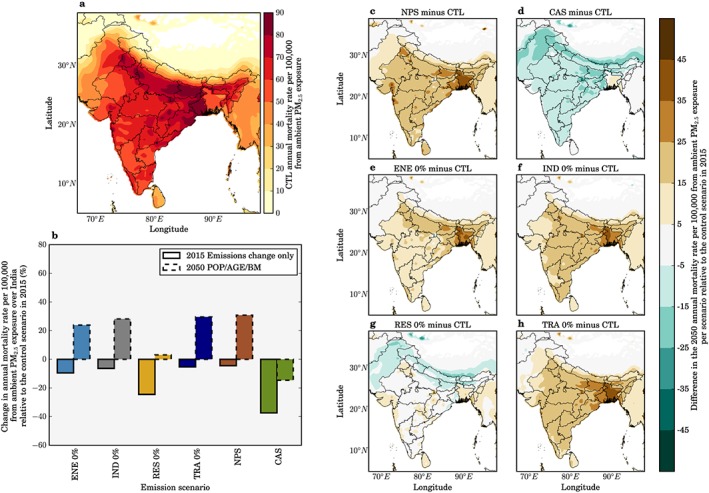
The impact of scenarios on annual mortality rate per 100,000 populations from exposure to ambient PM_2.5_ in India. (a) Annual mortality rate per 100,000 populations from exposure to ambient PM_2.5_ in India in 2015 from the control scenario. (b) National mean changes in annual mortality rate per 100,000 population estimates from ambient PM_2.5_ exposure per scenario, for both emissions only changes in 2015 and overall changes in 2050. (c–h) Difference in the annual mortality rate per 100,000 populations from ambient PM_2.5_ exposure in 2050 for IEA New Policy Scenario (NPS), IEA Clean Air Scenario (CAS), removal of power generation emissions (ENE 0%), removal of industry emissions (IND 0%), removal of residential energy use emissions (RES 0%), and removal of land transport emissions (TRA 0%) scenarios relative to the control scenario in 2015.

Figure 5 shows the impacts of the scenarios on the mortality rate per 100,000 populations from exposure to ambient PM_2.5_ across India. Under no change in emissions to 2050, the mortality rate increases by 39% to 86 deaths per 100,000 populations relative to 2015. The mortality rate is independent of population size, hence removing the changes from population growth. In 2050, the NPS and CAS change the mortality rate per 100,000 populations by +31% and −15%, respectively. In summary, the CAS stands out as the most effective scenario to reduce ambient PM_2.5_ concentrations across India, reducing the mortality rate per 100,000 populations in 2050 below 2015 control levels by 15%, while still increasing the total premature mortality estimate in 2050 above 2015 control levels by 7%. This highlights the challenge facing efforts to improve air quality‐related public health in India.

In all states except Delhi, residential energy use is the dominant sectoral contributor to ambient PM_2.5_ concentrations in the 2015 control scenario. In simulations where emissions from RES, ENE, IND, and TRA sectors are removed in 2050, the total premature mortality increases by 30%, 59%, 64%, and 68%, respectively, relative to the control scenario estimate for 2015 (Figure 4b). The increase in the total premature mortality estimate in 2050 due to population aging and growth can be offset by 407,000 (60%), 142,000 (21%), 105,000 (16%), and 68,000 (10%) premature mortalities by removing RES, ENE, IND, and TRA, respectively. The mean mortality rate per 100,000 populations across India in 2050 relative to 2015 increases by 3%, 24%, 29%, and 31% by removing RES, ENE, IND, and TRA, respectively (Figure 5b). In Delhi, multiple emission sources contribute strongly to the very high annual mean ambient PM_2.5_ concentrations of 122 μg/m^3^ in the 2015 control scenario, and although emissions from TRA dominate (36%), emissions from RES (27%), ENE (26%), and IND (13%) also contribute substantially. In West Bengal, annual mean ambient PM_2.5_ concentrations are also very high at 94 μg/m^3^; however, RES emissions heavily dominate the source contribution (62%), and the scenario removing these is equivalent to 75% of the potential health benefits from the CAS. In West Bengal, annual premature mortalities for the NPS and CAS in 2050 relative to the control scenario for 2015 are increased in line with the national averages of 68% and 7%, respectively. While for Delhi, both scenarios are less effective at reducing the disease burden where there is a 70% increase for the NPS and 22% increase for the CAS.

**Figure 6:**
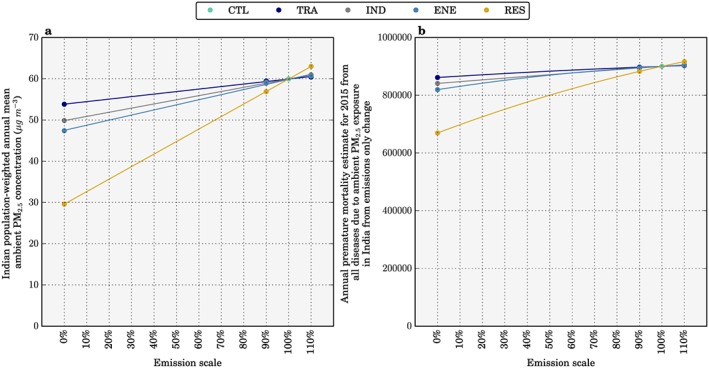
(a) The impact of emission scaling on population‐weighted annual mean ambient PM_2.5_ concentrations and (b) total annual premature mortality from exposure to ambient PM_2.5_ concentrations in India.

Figure 6 shows the impact of emission scaling from the idealized simulations (0%, −10%, and +10%) on population‐weighted annual mean ambient PM_2.5_ concentrations and the associated disease burden. There is an approximately linear reduction in population‐weighted ambient PM_2.5_ concentrations relative to individual source emission changes. However, there is a nonlinear reduction in premature mortality from the individual source change scenarios, due to the nonlinear exposure‐response function. Reductions in population‐weighted ambient PM_2.5_ concentrations are relatively larger than reductions in the associated disease burden.

### 3.3 Sensitivities to Demography and Baseline Mortality Rates

**Figure 7:**
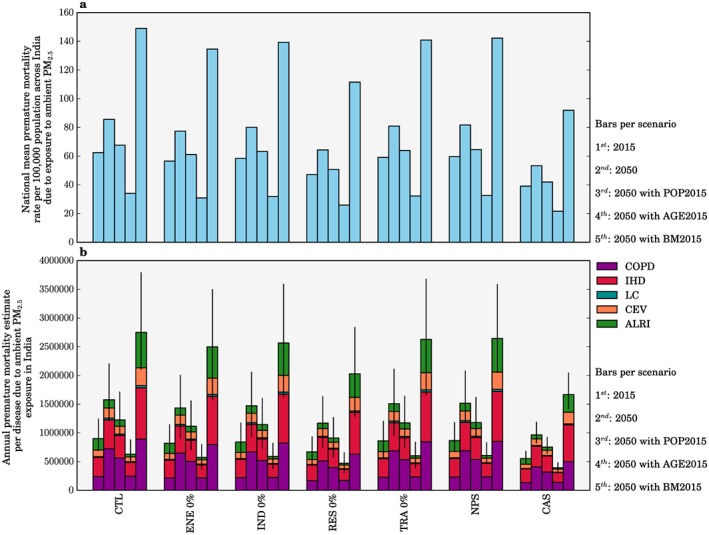
The impact of scenarios on the disease burden from exposure to ambient PM_2.5_ in India. (a) National mean annual premature mortality rate per 100,000 populations due to ambient exposure to PM_2.5_ in India. (b) Disease breakdown of health burden from ambient PM_2.5_ exposure in India. For each panel the bars show estimates for 2015, 2050, and 2050 with population density from 2015 to 2050, with population age grouping from 2015, and 2050, and with baseline mortality rates from 2015 (first to fifth bars per scenario). The vertical error bars show 95% uncertainty intervals (95UI) calculated from combining fractional errors in quadrature (see section 2).

We performed sensitivity studies estimating the disease burden from emission only changes in 2015, and then for 2050 individually using population density from 2015 (POP2015), population age groupings from 2015 (AGE2015), or baseline mortality rates from 2015 (BM2015) to explore the impact of each variable in turn. Figure 7a shows the change in the national mean premature mortality rate per 100,000 populations in India due to ambient PM_2.5_ exposure from each scenario. Figure 7b shows the disease breakdown of the total premature mortality estimates per scenario. Figures 7a and 7b do not show results from the −10% and +10% emission change scenarios as premature mortality estimates and mortality rates only change by ±2% for these scenarios. For emission‐only changes in 2015, the NPS and CAS reduced premature mortality estimates by 4% and 39%, respectively, while the individual removal of RES, ENE, IND, and TRA emissions reduced premature mortality estimates by 26%, 9%, 7%, and 4%, respectively (Figure 7b). Emission reductions have a larger impact through reducing respiratory diseases (ALRI and COPD), which respond more linearly, rather than the cardiovascular diseases (IHD and CEV), which have a more nonlinear response to changes in PM_2.5_ concentrations. This non‐linearity was also found in previous studies (Apte et al., 2015; Conibear et al., 2018; Kodros et al., 2016). These scenarios in which only emissions are modified highlight the need for stringent air quality management to reduce the disease burden in the highly polluted country of India due to the nonlinear exposure‐response function.

Each sensitivity (POP2015, AGE2015, and BM2015) uses 2015 data for that specific variable with 2050 data for the other variables. Each sensitivity therefore shows the influence of the other parameters in combination in 2050, compared with 2015. The difference between each sensitivity mortality estimate for 2050 and the control mortality estimate for 2015 is the impact of the temporal change (2050 minus 2015) in that specific variable. For the control scenario, premature mortality estimates in 2050 for POP2015, AGE2015, and BM2015 changed by −22%, −60%, and +74%, respectively, relative to control scenario estimates for 2050 (Figure 7). Consequently, population aging and growth together through to 2050 in India increase the number of people susceptible to air pollution, while a decrease in baseline mortality rates offsets a large part of this increase. The large sensitivity of premature mortality estimates to population aging and baseline mortality rates illustrates the importance of demographic and epidemiological transitions in the future disease burden from exposure to ambient PM_2.5_, which was also found in recent previous studies (Chowdhury et al., 2018; GBD MAPS Working Group, 2018).

### 3.4 Comparison to Previous Studies

**Figure 8:**
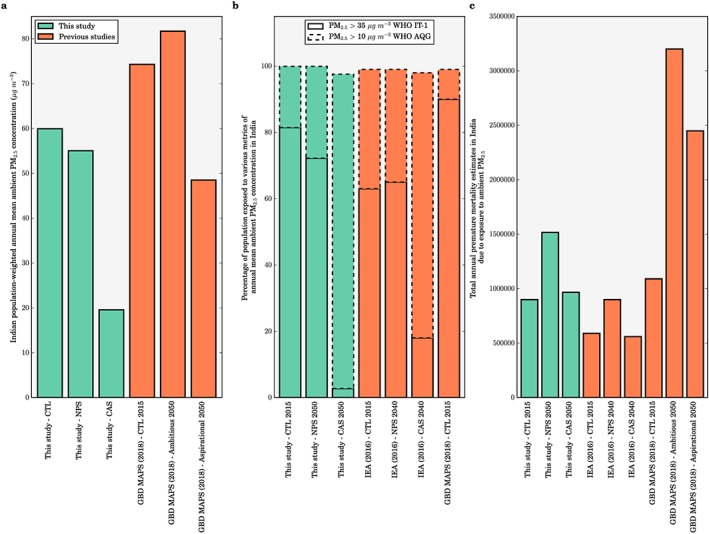
Comparison of the impacts of different scenarios on ambient PM_2.5_ concentrations and the associated disease burden in India from this study with previous studies. (a) Comparison of population‐weighted annual mean ambient PM_2.5_ concentrations. (b) Comparison of percentage of the population exposed to various metrics of annual mean ambient PM_2.5_ concentrations in India. (c) Comparison of total premature mortality estimates due to exposure to ambient PM_2.5_ per year in India from different scenarios.

Figure 8 compares our simulated impacts of the different scenarios on ambient PM_2.5_ concentrations and the associated disease burden in India with previous studies (GBD MAPS Working Group, 2018; International Energy Agency, 2016a). We estimate a larger percentage of the population exposed to ambient PM_2.5_ concentrations exceeding the WHO IT‐1 compared to the IEA study (International Energy Agency, 2016a; Figure 8b). The lower estimate in the IEA study could potentially be a reflection of lower spatial resolution used in the IEA study underestimating the high ambient PM_2.5_ concentrations in India, in addition to the different modeling choices where the IEA study used the Greenhouse gas – Air pollution Interactions and Synergies (GAINS) model (Amann et al., 2011) driven by the European Monitoring and Evaluation Programme (EMEP) chemical transport model (Simpson et al., 2012). The control scenario estimate of total premature mortality in 2015 from the IEA study was 34% smaller than our study (Figure 8c). The lower estimate is likely due to the combination of lower ambient PM_2.5_ concentrations, older baseline mortality rates (2011 versus 2016 in our study) corrected for risk but not cause of mortality, and the use of older exposure‐response functions from the GBD2013 (GBD 2013 Risk Factors Collaborators, 2015). The GBD2013 exposure‐response functions have weaker relationships between risk and ambient PM_2.5_ concentrations relative to the updated exposure‐response functions from the GBD2016 (Cohen et al., 2017; GBD 2016 Risk Factors Collaborators, 2017) as used by our study and the GBD MAPS Working Group study. The IEA study estimated slightly smaller changes in premature mortality of +53% and −5% for the NPS and CAS, respectively, relative to control scenario in 2015 compared to our estimates of +68% and +7%, primarily due to an earlier year of estimation (2040 versus 2050 in our study) reducing the impacts of population growth and aging.

The control scenario population‐weighted annual mean ambient PM_2.5_ concentrations from the GBD MAPS Working Group study (GBD MAPS Working Group, 2018) were 24% larger than those in tour study (Figure 8a), potentially due to their use of higher resolution (0.1° × 0.1°) modeling and data assimilation within a Bayesian hierarchical model in the control scenario. The fractional impacts of the scenarios on ambient PM_2.5_ concentrations were then derived using the South Asia nested version of the Goddard Earth Observing System (GEOS)‐Chem chemical transport model at coarser resolution (0.5° × 0.67°) to scale the higher resolution ambient PM_2.5_ concentrations. The ambitious and aspirational scenarios analyzed in the GBD MAPS Working Group study are different to the NPS and CAS scenarios analyzed in our study. Both the IEA NPS and GBD MAPS Working Group ambitious scenarios are based on relevant existing and planned policies as of 2016 including India's Intended Nationally Defined Contributions. However, the technology shifts and sectoral growth rates vary between the different scenarios. In the NPS SO_2_, NO_x_, and PM_2.5_ emissions increase by 10%, 10%, and 7% in 2040 relative to 2015, respectively, while in the GBD MAPS Working Group ambitious scenario SO_2_, NO_x_, and PM_2.5_ emissions increase by 156%, 96%, and 26% in 2050 relative to 2015, respectively. Similarly, while both the IEA CAS and the GBD MAPS Working Group aspirational scenario represent stringent air quality management, the specific realizations of these scenarios vary between the studies. In the CAS SO_2_, NO_x_, and PM_2.5_ emissions change by −69%, −50%, and −76% in 2040 relative to 2015 respectively, while in the GBD MAPS Working Group aspirational scenario SO_2_, NO_x_, and PM_2.5_ emissions change by −6%, +13%, and −67% in 2050 relative to 2015, respectively. The GBD MAPS Working Group found larger resulting ambient PM_2.5_ concentrations from their scenarios (Figure 8a) and consequent increases in premature mortality in 2050 (Figure 8c). Overall, our study, the GBD MAPS Working Group study, and the IEA study all find large potential public health benefits from stringent air quality management relative to a business‐as‐usual scenario.

Previous research has found the future impacts of climate change on PM_2.5_ concentrations and associated mortality in India to be substantially smaller than the impacts from emission changes. Specifically, two recent studies analyzed the combined impacts of climate change and emission scenarios on future air quality in South Asia by 2050 (Kumar et al., 2018; Pommier et al., 2018). Pommier et al. (2018) found the large increase in anthropogenic emissions in India by 2050 to have an order of magnitude larger impact on PM_2.5_ concentrations than the impacts of climate change. Kumar et al. (2018) used the same complex aerosol model (MOSAIC) as our study and found South Asian PM_2.5_ concentrations to change by +13 and +1 μg/m^3^ by 2050 relative to 2015 under Representative Concentration Pathways (RCP) 8.5 and RCP6.0, respectively. Kumar et al. (2018) then qualitatively related changes in meteorological variables with those in air quality due the limited number of simulations conducted relative to the large ensemble of simulations required to quantify the impacts from climate change. The health impacts of climate changes on PM_2.5_ concentrations in India were estimated by Silva et al. (2017) to be 80,200 deaths per year by 2100. This is 3% of the increase in PM_2.5_ exposure associated mortality in India by 2050 due to emission changes under a reference scenario (GBD MAPS Working Group, 2018). Both previous studies estimating the future health impacts from air pollution in India under different scenarios also used fixed meteorology to focus on the impacts from different air pollution control pathways (GBD MAPS Working Group, 2018; International Energy Agency, 2016a).

Air quality is also impacted by transport of pollution from distant sources (TF HTAP, 2010). In 2007, PM_2.5_ produced inside India was associated with 75,000 premature deaths outside of India, while PM_2.5_ produced outside India was associated with 67,000 premature deaths inside India (Zhang et al., 2017). Aerosol transport from Africa and the Middle East is associated with 83,000 and 77,000 premature mortalities in India, primarily from dust (Liu et al., 2009). Regional transport of PM_2.5_ into South Asia contributes 7% of the total mortality impact (Anenberg et al., 2014). The future contribution of regional transport to air pollution and associated disease burden in India may change under different scenarios and is an important area of future research.

Our study, in agreement with previous studies (GBD MAPS Working Group, 2018; International Energy Agency, 2016a), finds large increases in the future disease burden associated with ambient PM_2.5_ exposure in India due to population growth and aging. New policies as of 2016 or small (−10%) changes in emissions provide only small improvements in air quality and public health, where the impacts are heavily outweighed by the demographic transition. Stringent air quality management, such as under the IEA CAS, removing residential energy use emissions, or the GBD MAPS Working Group aspirational scenario will be required to provide large improvements in air quality and important public health benefits. The removal of residential energy use and land transport emissions might have further health benefits if exposures are studied at finer scales due to the collocation of emissions with exposures. The changes in disease burden estimates from ambient PM_2.5_ exposure do not consider that for some scenarios there is a large accompanying reduction in the disease burden from reducing household air pollution from solid fuel use (e.g., the CAS and RES 0%).

## 4 Conclusion

Exposure to ambient particulate matter is a leading risk factor for public health in India. Business‐as‐usual economic and industrial growth in India to 2050 is predicted to increase emissions and further worsen ambient PM_2.5_ concentrations. Previous studies of alternative air pollution control scenarios in India have used relatively coarse spatial resolution models to estimate the impacts of the scenarios to ambient PM_2.5_ concentrations. In this study, we use a high‐resolution online‐coupled model and the latest exposure‐response function to estimate the impacts of multiple Indian emission scenarios to ambient PM_2.5_ concentrations and human health in India. We do not include impacts of climate change or the impacts of changing emissions from outside India. We find that with no emissions growth in India, the disease burden from exposure to ambient PM_2.5_ in 2050 will increase by 75% relative to 2015 due to population aging and growth increasing the number of people susceptible to air pollution, partly offset by decreasing baseline mortality rates. We estimate that the International Energy Agencies NPS and CAS in 2050 can reduce population‐weighted ambient PM_2.5_ concentrations below 2015 levels by 9% and 68%, respectively. These reductions in ambient PM_2.5_ concentrations reduce the annual premature mortality estimate by 61,000 and 610,000 deaths, which is 9% and 91% of the projected increase in premature mortalities due to population growth and aging. Throughout India, the CAS stands out as the most effective scenario to reduce ambient PM_2.5_ concentrations and the associated disease burden, reducing the 2050 mortality rate per 100,000 below 2015 control levels by 15%. However, even under the strong emission reductions of the CAS, population growth and aging mean that the annual premature mortality in 2050 will be 7% greater than in 2015. Our results show that small emission changes bring small improvements to air quality and human health, where the impacts are heavily outweighed by the demographic transition to 2050. Strict implementation of air quality management, such as under the IEA CAS or removing residential energy use emissions from solid fuel use, can reduce the substantial and increasing health impacts from air pollution exposure in India bringing important public health benefits.

## Conflict of Interest

The authors declare no conflicts of interest relevant to this study.

## Supporting information

Supporting Information S1

Data Set S1
