# Supplementary material for: Stringent Emission Control Policies Can Provide Large Improvements in Air Quality and Public Health in India
Source: Geohealth. 2018 Jul 3;2(7):196–211. doi: 10.1029/2018GH000139 (PMC7203661; doi:10.1029/2018GH000139)
Supplement: Supplementary file 1 — Supporting Information S1 [file GH2-2-196-s001.pdf]

GeoHealth

Supporting information for

Stringent emission control policies can provide large improvements in air quality and public health in India

Luke Conibear[1,2], Edward W. Butt[2], Christoph Knote[3], Stephen R. Arnold[2] and Dominick V. Spracklen[2]

[1] Engineering and Physical Sciences Research Council (EPSRC) Centre for Doctoral Training (CDT) in Bioenergy, University of Leeds, Leeds, LS2 9JT, UK

[2] Institute for Climate and Atmospheric Science, School of Earth and Environment, University of Leeds, Leeds, LS2 9JT, UK

[3] Meteorological Institute, LMU Munich, Theresienstr. 37, 80333, Munich, Germany

Corresponding author: Luke Conibear (pmlac@leeds.ac.uk)

Contents

Supplementary Table 1: Model Setup and parameterisation used in the Weather Research and Forecasting model coupled with Chemistry (WRF-Chem) model.

Supplementary Table 2: Scaling factors per emission sector and air pollutant in India (International Energy Agency, 2016).

Supplementary Figure 1: Integrated-exposure response (IER) functions estimating relative risk (RR) of mortality from ambient $PM_{2.5}$ concentrations from Global Burden of Diseases, Injuries, and Risk Factors Study (GBD) 2016 (GBD 2016 Risk Factors Collaborators, 2017). Mean exposure-response shown in bold line for ischaemic heart disease (IHD), cerebrovascular disease (CEV), chronic obstructive pulmonary disease (COPD), acute lower respiratory infections (ALRI) and lung cancer (LC). IHD and CEV have shaded regions representing the variation with age groups.

Supplementary Figure 2: Variation in baseline mortality, population age distribution and population density for India between 2015 and 2050 from the International Futures (IFs) integrated modelling system (Hughes et al., 2011) baseline scenario. Baseline mortality rates for a) acute lower respiratory infections (ALRI), b) chronic obstructive pulmonary disease (COPD), c) ischaemic heart disease (IHD), d) cerebrovascular disease (CEV), and e) lung cancer (LC) in 2015 and 2050. f Population age distribution in 2015 and 2050. g Spatial distribution of population density in 2015 for South Asia. h Difference in population density for South Asia between 2050 and 2015.

Supplementary Figure 3: Variation in baseline mortality, population age distribution and population density for India in 2015 between the International Futures (IFs) integrated modelling system (Hughes et al., 2011) baseline scenario and data used in GBD2016 (GBD 2016 Risk Factors Collaborators, 2017). Baseline mortality rates for a) acute lower respiratory infections (ALRI), b) chronic obstructive pulmonary disease (COPD), c) ischaemic heart

disease (IHD), d) cerebrovascular disease (CEV), and e) lung cancer (LC) in 2015 from IFs and GBD2016 (Institute for Health Metrics and Evaluation, 2018). Note that for ALRI, IFs provides age group 0-4 while GBD2016 provides age group 0-5. f Population age distribution in 2015 from IFs and GBD2016 (Global Burden of Disease Study 2016, 2017). g Difference in population density for South Asia in 2015 from IFs and Gridded Population of the World, Version 4 (GPWv4) (Center for International Earth Science Information Network & NASA Socioeconomic Data and Applications Center, 2016).

Additional Supporting Information (Files uploaded separately)

Supplementary data containing results per Indian state per scenario.

Supplementary Table 1: Model Setup and parameterisation used in the Weather Research and Forecasting model coupled with Chemistry (WRF-Chem) model.

**Model Setup and Parameterisation**	
**Process**	**Method**
**Domain**	60° to 100° East, 0° to 40° North
**Timestep**	180 seconds, with Runge-Kutta 2nd and 3rd order time integration
**Horizontal**	Resolution of 30 km along a 140x140 grid, with Arakawa C-grid staggering and 2nd to 6th order advection schemes
**Vertical**	27 vertical levels (top at 10 hPa) with terrain-following hydrostatic pressure coordinates and 2nd to 6th order advection schemes
**Precipitation microphysics**	Thompson scheme (Thompson et al., 2008)
**Longwave radiation**	RRTM longwave (Mlawer et al., 1997), called every 30 mins
**Shortwave radiation**	RRTM shortwave (Pincus et al., 2003), called every 30 mins
**Boundary layer physics**	Mellor-Yamada Nakanishi and Niino 2.5 (Nakanishi et al., 2006), called every timestep
**Land surface**	Noah Land Surface Model (Ek et al., 2003)
**Convective parameterisation**	Grell 3-D ensemble (Grell et al., 2002), called every 60 seconds
**Gas-phase chemistry scheme**	MOZART-4 using KPP (Emmons et al., 2010), chem_opt=201 (Hodzic & Knote, 2014), called every 12 mins
**Photolysis scheme**	Madronich fTUV (Tie et al., 2003), called every 30 mins
**Aerosol scheme**	MOSAIC 4-bin (Zaveri et al., 2008), called every 12 mins
**Dust**	GOCART online with AFWA, dust_opt=3 (Chin et al., 2000, 2002)
**Initial & boundary chemistry/aerosol**	MOZART-4/GEOS5 (NCAR, 2016)
**Initial & boundary meteorology**	NCEP GFS and NCEP FNL (NCEP et al., 2000, 2007)

Supplementary Table 2: Scaling factors per emission sector and air pollutant in India (International Energy Agency, 2016).

	New Policy Scenario (NPS)			Clean Air Scenario (CAS)		
	SO_2	NOx	$PM_{2.5}$	SO_2	NOx	$PM_{2.5}$
**Industry (IND)**	2.46	2.75	2.52	0.61	1.24	0.48
**Power (ENE)**	0.11	0.73	0.21	0.05	0.40	0.07
**Residential (RES)**	0.55	1.10	0.61	0.37	0.54	0.15
**Transport (TRA)**	2.20	0.55	0.53	1.12	0.22	0.43
**Other**	1.84	1.10	1.07	0.75	0.27	0.00
**Total**	1.10	1.10	1.07	0.31	0.50	0.24

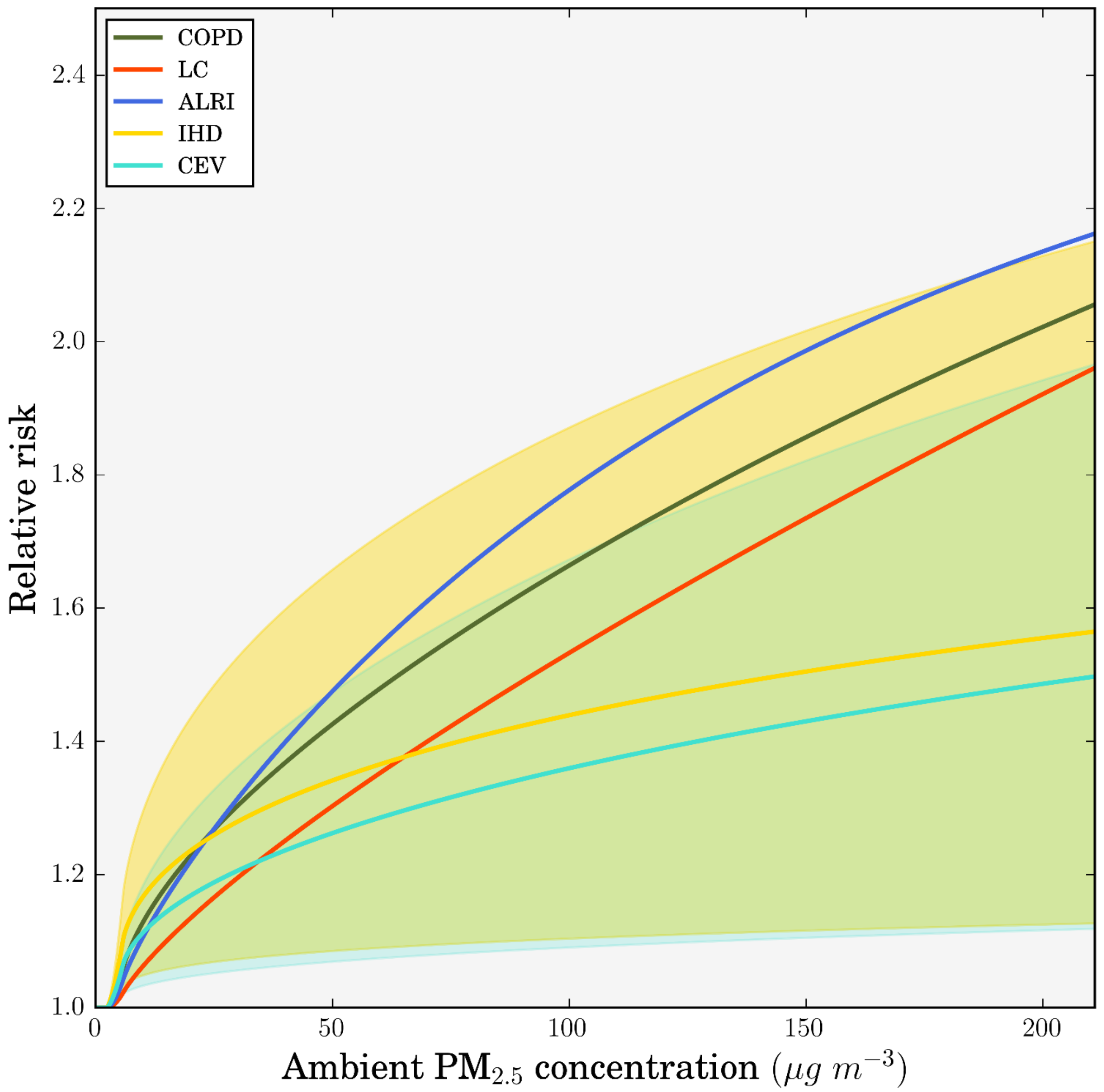

Supplementary Figure 1: Integrated-exposure response (IER) functions estimating relative risk (RR) of mortality from ambient $PM_{2.5}$ concentrations from Global Burden of Diseases, Injuries, and Risk Factors Study (GBD) 2016 (GBD 2016 Risk Factors Collaborators, 2017). Mean exposure-response shown in bold line for ischaemic heart disease (IHD), cerebrovascular disease (CEV), chronic obstructive pulmonary disease (COPD), acute lower respiratory infections (ALRI) and lung cancer (LC). IHD and CEV have shaded regions representing the variation with age groups.

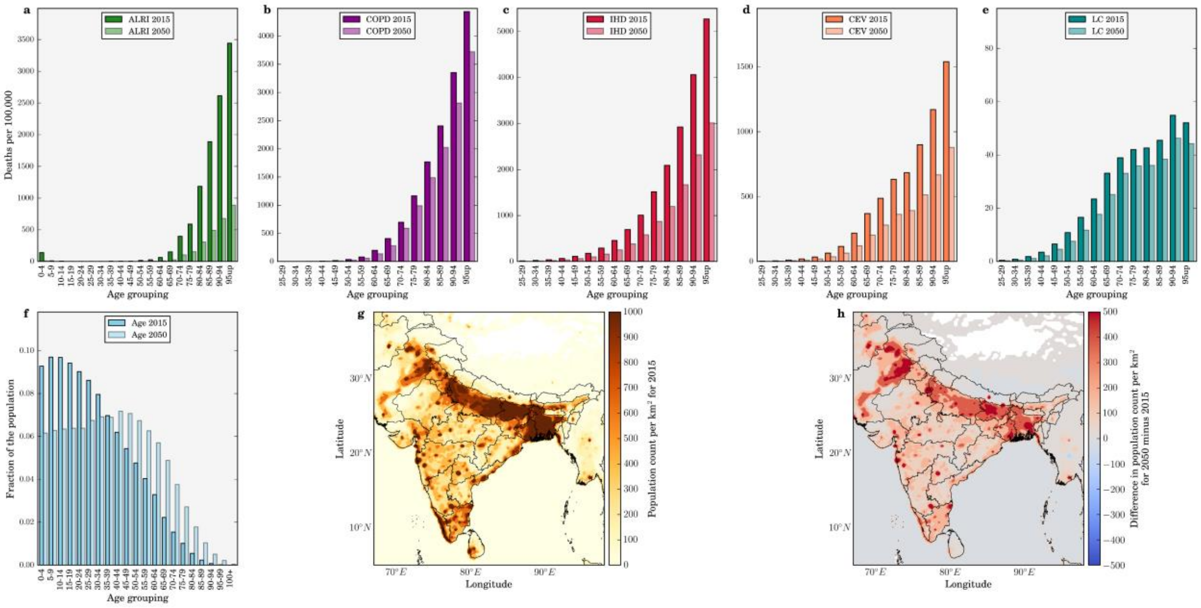

Supplementary Figure 2: Variation in baseline mortality, population age distribution and population density for India between 2015 and 2050 from the International Futures (IFs) integrated modelling system (Hughes et al., 2011) baseline scenario. Baseline mortality rates for **a)** acute lower respiratory infections (ALRI), **b)** chronic obstructive pulmonary disease (COPD), **c)** ischaemic heart disease (IHD), **d)** cerebrovascular disease (CEV), and **e)** lung cancer (LC) in 2015 and 2050. **f** Population age distribution in 2015 and 2050. **g** Spatial distribution of population density in 2015 for South Asia. **h** Difference in population density for South Asia between 2050 and 2015.

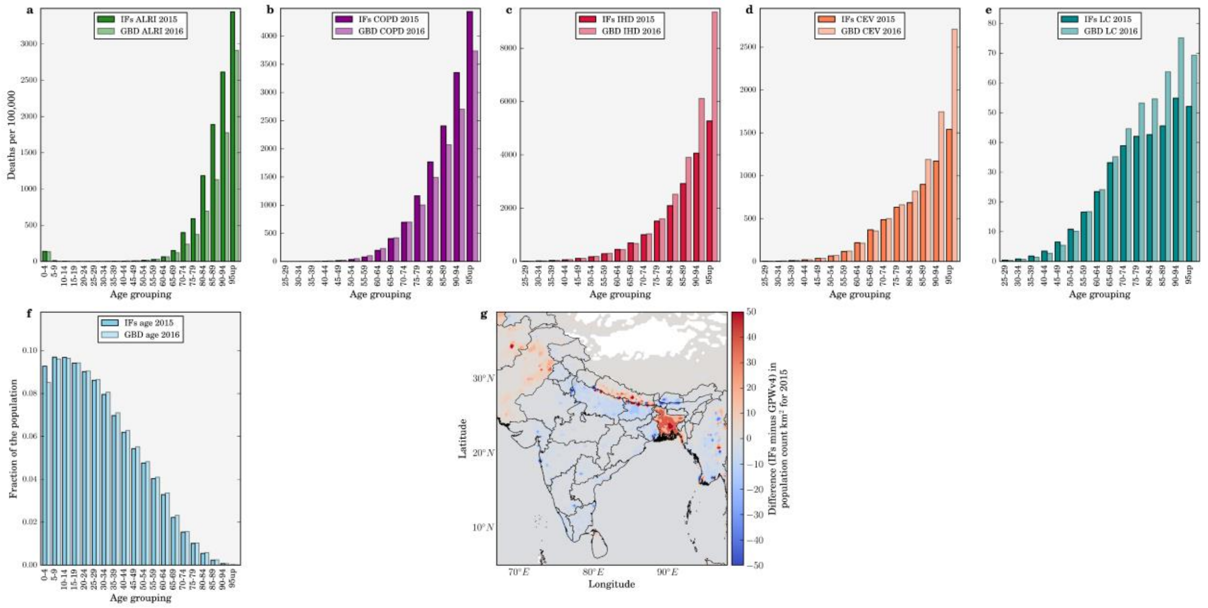

Supplementary Figure 3: Variation in baseline mortality, population age distribution and population density for India in 2015 between the International Futures (IFs) integrated modelling system (Hughes et al., 2011) baseline scenario and data used in GBD2016 (GBD 2016 Risk Factors Collaborators, 2017). Baseline mortality rates for **a)** acute lower respiratory infections (ALRI), **b)** chronic obstructive pulmonary disease (COPD), **c)** ischaemic heart disease (IHD), **d)** cerebrovascular disease (CEV), and **e)** lung cancer (LC) in 2015 from IFs and GBD2016 (Institute for Health Metrics and Evaluation, 2018). Note that for ALRI, IFs provides age group 0-4 while GBD2016 provides age group 0-5. **f** Population age distribution in 2015 from IFs and GBD2016 (Global Burden of Disease Study 2016, 2017). **g** Difference in population density for South Asia in 2015 from IFs and Gridded Population of the World, Version 4 (GPWv4) (Center for International Earth Science Information Network & NASA Socioeconomic Data and Applications Center, 2016).

References

Center for International Earth Science Information Network, & NASA Socioeconomic Data and Applications Center. (2016). Gridded Population of the World, Version 4 (GPWv4): Population Density. https://doi.org//10.7927/H4NP22DQ

Chin, M., Ginoux, P., Kinne, S., Torres, O., Holben, B. N., Duncan, B. N., et al. (2002). Tropospheric Aerosol Optical Thickness from the GOCART Model and Comparisons with Satellite and Sun Photometer Measurements. *Journal of the Atmospheric Sciences*, *59*(3), 461–483. https://doi.org/10.1175/1520-0469(2002)059

Chin, M., Rood, R. B., Lin, S.-J., Müller, J.-F., & Thompson, A. M. (2000). Atmospheric sulfur cycle simulated in the global model GOCART: Model description and global properties. *Journal of Geophysical Research*, *105*(D20), 24671–24687. https://doi.org/10.1029/2000JD900384

Ek, M. B., Mitchell, K. E., Lin, Y., Rogers, E., Grunmann, P., Koren, V., et al. (2003). Implementation of Noah land surface model advances in the National Centers for Environmental Prediction operational mesoscale Eta model. *Journal of Geophysical Research Atmospheres*, *108*(D22), 8851–8867. https://doi.org/10.1029/2002JD003296

Emmons, L. K., Walters, S., Hess, P. G., Lamarque, J.-F., Pfister, G. G., Fillmore, D., et al. (2010). Description and evaluation of the Model for Ozone and Related chemical Tracers, version 4 (MOZART-4). *Geoscientific Model Development Discussions*, (2), 43–67. https://doi.org/10.5194/gmdd-2-1157-2009

GBD 2016 Risk Factors Collaborators. (2017). Global, regional, and national comparative risk assessment of 84 behavioural, environmental and occupational, and metabolic risks or clusters of risks, 1990–2016: a systematic analysis for the Global Burden of Disease Study 2016. *The Lancet*, *390*, 1345–1422. https://doi.org/10.1016/S0140-6736(17)32366-8

Global Burden of Disease Study 2016. (2017). Global Burden of Disease Study 2016 (GBD 2016) Population Estimates 1950-2016. Retrieved from http://ghdx.healthdata.org/record/global-burden-disease-study-2016-gbd-2016-population-estimates-1950-2016

Grell, G. A., & Devenyi, D. (2002). A generalized approach to parameterizing convection combining ensemble and data assimilation techniques. *Geophysical Research Letters*, *29*(14), 10–13. https://doi.org/10.1029/2002GL015311

Hodzic, A., & Knote, C. (2014). WRF-Chem 3.6.1: MOZART gas-phase chemistry with MOSAIC aerosols. *Atmospheric Chemistry Division (ACD), National Center for Atmospheric Research (NCAR)*, 7.

Hughes, B. B., Kuhn, R., Peterson, C. M., Rothman, D. S., Solórzano, J. R., Mathers, C. D., & Dickson, J. R. (2011). Projections of global health outcomes from 2005 to 2060 using the International Futures integrated forecasting model. *Bulletin of the World Health Organization*, *89*(7), 478–486. https://doi.org/10.2471/BLT.10.083766

Institute for Health Metrics and Evaluation. (2018). GBD Compare Data Visualization. Retrieved February 13, 2018, from vizhub.healthdata.org/gbd-compare

International Energy Agency. (2016). *Energy and Air Pollution. World Energy Outlook Special Report*. Paris, France.

Mlawer, E. J., Taubman, S. J., Brown, P. D., Iacono, M. J., & Clough, S. A. (1997). Radiative transfer for inhomogeneous atmospheres: RRTM, a validated correlated-k model for the

longwave. *Journal of Geophysical Research*, *102*(D14), 16663–16682. https://doi.org/10.1029/97JD00237

Nakanishi, M., & Niino, H. (2006). An improved Mellor-Yamada Level-3 model: Its numerical stability and application to a regional prediction of advection fog. *Boundary-Layer Meteorology*, *119*(2), 397–407. https://doi.org/10.1007/s10546-005-9030-8

NCAR. (2016). ACOM MOZART-4/GEOS-5 global model output. *UCAR*. Retrieved from http://www.acom.ucar.edu/wrf-chem/mozart.shtml

NCEP, National Weather Service, NOAA, & U.S. Department of Commerce. (2000). NCEP Final (FNL) Operational Model Global Tropospheric Analyses, continuing from July 1999. Research Data Archive at the National Center for Atmospheric Research, Computational and Information Systems Laboratory. https://doi.org/http://dx.doi.org/10.5065/D6M043C6.

NCEP, National Weather Service, NOAA, & U.S. Department of Commerce. (2007). NCEP Global Forecast System (GFS) Analyses and Forecasts. Research Data Archive at the National Center for Atmospheric Research, Computational and Information Systems Laboratory. https://doi.org/http://rda.ucar.edu/datasets/ds084.6/

Pincus, R., Barker, H. W., & Morcrette, J.-J. (2003). A fast, flexible, approximate technique for computing radiative transfer in inhomogeneous cloud fields. *Journal of Geophysical Research*, *108*(D13), 1–5. https://doi.org/10.1029/2002JD003322

Thompson, G., Rasmussen, R. M., & Manning, K. (2008). Explicit Forecasts of Winter Precipitation Using an Improved Bulk Microphysics Scheme. Part II: Implementation of a New Snow Parameterization. *American Meteorological Society*, *136*(2), 5095–5115. 10.1175/2008MWR2387.1

Tie, X., Madronich, S., Walters, S., Zhang, R., Rasch, P., & Collins, W. (2003). Effect of clouds on photolysis and oxidants in the troposphere. *Journal of Geophysical Research*, *108*(D20), 4642, 1–11. https://doi.org/10.1029/2003JD003659

Zaveri, R. A., Easter, R. C., Fast, J. D., & Peters, L. K. (2008). Model for Simulating Aerosol Interactions and Chemistry (MOSAIC). *Journal of Geophysical Research Atmospheres*, *113*(13), 1–29. https://doi.org/10.1029/2007JD008782
